# The functional connectivity between dorsal-medial prefrontal cortex and middle cingulate cortex links trait rumination and depressive symptoms

**DOI:** 10.1371/journal.pone.0328895

**Published:** 2025-08-04

**Authors:** Yinghui Guo, Dongtao Wei

**Affiliations:** 1 School of Public Policy and Administration, Chongqing University, Chongqing, China; 2 Public Economic and Public Policy Research Center, Chongqing University, Chongqing, China; 3 Faculty of Psychology, Southwest University, Chongqing, China; 4 China Key Laboratory of Cognition and Personality (Ministry of Education), Southwest University, Chongqing, China; University of Pittsburgh School of Medicine, UNITED STATES OF AMERICA

## Abstract

Task-based functional neuroimaging has revealed that rumination is associated with functional activations in the default mode network (DMN). The present study aimed to examine whether resting state functional connectivity (FC) within the DMN is associated with individual differences in trait rumination. Using the seed-based functional connectivity analysis in a relatively large sample of late adolescents, this study investigated the neural correlates of trait rumination and their associations with depressive symptoms. Results showed that higher functional connectivity between the left dorsal medial prefrontal cortex (DMPFC) and middle cingulate cortex (MCC) was positively associated with trait rumination. Additionally, stronger FC between the left DMPFC and the right inferior parietal lobe (IPL) was also related to trait rumination. Furthermore, logistic regression analysis indicated that FC strength between the left DMPFC and MCC was significantly associated with depressive symptom severity. These findings suggest DMPFC-related network may reflect neural mechanisms linked to both trait rumination and depressive symptoms in late adolescence.

## 1. Introduction

Rumination, defined as a repetitive and passive focus on negative emotions and their causes, is a well-established cognitive risk factor for the onset, persistence, and severity of depression [1–4]. The response style theory proposed by Nolen-Hoeksema [5] has inspired extensive research into the cognitive and neural mechanisms of rumination. Trait rumination, in particular, reflects a stable tendency toward this thinking style and has been associated with maladaptive processes such as impaired cognitive control [6], excessive attention to negative stimuli [5,7], distorted self-referential processing [8], and altered autobiographical memory recall [9].

Given that many of these cognitive functions are supported by the default mode network (DMN), it is unsurprising that a growing body of neuroimaging research has implicated the DMN in rumination. Meta-analyses have revealed that patients with major depressive disorder (MDD) [10,11] as well as healthy individuals [12], show abnormal DMN activation or connectivity during ruminative thought. Task-based studies further demonstrate that DMN regions, including the medial prefrontal cortex (MPFC), posterior cingulate cortex (PCC), and precuneus, are more active during rumination than during distraction tasks that promote externally directed attention [13–16]. Resting-state fMRI studies examining trait rumination have yielded mixed results regarding DMN functional connectivity (FC). Some studies report increased FC within DMN regions [17,18], while others suggest decreased or disrupted connectivity [19]. These inconsistencies may stem from differences in sample characteristics (e.g., clinical vs. non-clinical), developmental stage, or methodological approaches. For instance, DMN connectivity patterns may differ across adolescence and adulthood or vary depending on illness chronicity. Therefore, focusing on late adolescents, a critical developmental stage for the emergence of both rumination and depression, may help clarify these discrepancies.

Previous research on major depressive disorder (MDD) has reported both hyperconnectivity and hypoconnectivity within the default mode network (DMN), particularly highlighting inconsistencies in functional connectivity (FC) findings. Notably, several studies have identified increased FC within the anterior DMN, including regions such as the dorsomedial prefrontal cortex (DMPFC), ventromedial prefrontal cortex (VMPFC), pregenual anterior cingulate cortex (pgACC), and medial orbitofrontal cortex (OFC), in patients with MDD [20–22]. These anterior regions are consistently associated with self-referential thinking and emotional processing, which are core features of rumination. For example, Zhu et al. used independent component analysis to reveal increased connectivity within the anterior DMN in MDD patients [20], a finding replicated in other studies [21,22]. In contrast, Yan et al. conducted a large-sample resting-state study and found decreased FC within the DMN among individuals with recurrent MDD, with lower FC levels correlating with higher depressive symptom severity [23]. Similarly, studies focusing on specific regions of interest (ROI) have demonstrated that connectivity between the subgenual ACC (sgACC) and DMPFC is positively associated with depression severity in adolescents [24], and that patients with first-episode, medication-naïve MDD show increased homogeneity in the left DMPFC compared to healthy controls [25]. These seemingly contradictory results underscore the complexity of DMN functioning in depression. One possible explanation lies in differences in DMN subregions, as the anterior and posterior DMN may play distinct roles in self-focused and memory-related cognitive processes, respectively. Methodological factors such as illness stage (e.g., first-episode vs. recurrent), sample age, and analytic approach (e.g., ICA vs. seed-based FC) may also contribute to variability in findings. Importantly, the DMPFC, sometimes referred to as the “dorsal nexus” because of its extensive connectivity with affective and cognitive control regions, has emerged as a consistent site of altered connectivity in depression [26]. while other studies have reported decreased connectivity in recurrent MDD [23]. Such divergent findings highlight the importance of examining DMN subcomponents (e.g., anterior vs. posterior regions) and specifying seed regions when investigating trait rumination and depression risk. Of note, the dorsomedial prefrontal cortex (DMPFC), often implicated in self-referential thinking and social cognition, has been identified as a key hub in depression-related DMN alterations [25,26].

The present study aimed to investigate the potential neural mechanism underlying trait rumination during resting state and to examine their associations with depressive symptoms. Functional connectivity (FC) analysis is a commonly used approach to assess temporal correlations between spatially distinct brain regions [27] making it well suited for exploring large-scale network such as those within the default mode network [27]. A total of 715 healthy college students from Southwest University participated in this study. Based on prior evidence linking the medial prefrontal cortex (MPFC) to both rumination and depression, we selected the dorsomedial prefrontal cortex (DMPFC) and ventromedial prefrontal cortex (VMPFC), bilaterally, as regions of interest (ROIs). We hypothesized that increased resting-state functional connectivity of the MPFC would be associated with higher levels of trait rumination. Furthermore, we explored whether MPFC-related functional connectivity was also linked to depressive symptom severity.

## 2. Method

### 2.1. Participants

The sample employed in our study consists of data from two research projects, namely the Southwest University Longitudinal Imaging Multimodal (SLIM) project and Gene-Brain-Behavior (GBB) project. Participants were recruited between June 1, 2012 and December 27, 2015. All participants provided written informed consent prior to participation. For participants under the age of 18, parental consent was also obtained. The recruitment program and exclusion criteria are detailed in our previous publications [23].The former are available for research through the International Data-sharing Initiative(INDI, http://fcon_1000.projects.nitrc.org/indi/retro/southwestuni_qiu_index.html). The goal of the project was to investigate the associations among individual differences in brain structure and function, creativity, and mental health.

Following the exclusion of participants with missing demographic information (e.g., name, gender, and serial number) across different assessments and tasks, abnormal structural images (e.g., enlarged ventricles), poor functional imaging signal intensity, and excessive head motion, we preserved data from 136 participants in SLIM, 615 participants in GBB, aged 16–26 years old, whose mean age is 19.6 years old(SD = 1.48).The sample consisted of 331 males (44% of the total sample) and 420 females (56% of the total sample). All of the participants completed the Short Ruminative Response Scale(SRRS) and Beck Depression Inventory-II (BDI-II) and underwent a resting-state functional and structural magnetic resonance imaging (MRI) scan. Scinece all participants had passed their physical examinations during their freshman year, standard physical examinations were not conducted. Instead, a self-report questionnaire was used to assess their physical health. No participants in this study had a serious physical illness at the time of scanning.. To assess the potential mental disorders, two well-trained and experienced graduate students in the school of psychology performed the Structured Clinical Interview for the DSM-IV. The students did not meet the DSM-IV criteria for psychiatric disorders and did not use drugs that could affect brain function (including antidepressant drugs). None of them developed a psychiatric illness between the different scans. This study was approved by the Research Ethics Committee of the Brain Imaging Centre of Southwest University. Informed written consent was obtained from each subject. This study was conducted in accordance with the Declaration of Helsinki, revised in 1989.

### 2.2. Measurements

Demographic information and self-report data were completed during the adaptation phase of the laboratory visit. The 10-item Chinese Short Ruminative Responses Scale (SRRS) [28] was used to assess the degree of rumination, which was revised from the Ruminative Responses Scale (RRS) developed by Nolen-Hoeksema and has excellent reliability and validity [5,29]. The SRRS consists of two subscales: sensitive rumination and assessment rumination, which closely correspond to the constructs of brooding and reflection, respectively. Sample items include “I think about my tired and painful feelings” and “I think about how often I feel sad.” Participants rated the frequency of their ruminative responses on a 4-point Likert scale (1 = almost never, 4 = almost always). In our present sample, the scale had a high level of internal consistency (Cronbach’s α = 0.88).

The Beck Depression Inventory-II (BDI-II) is a 21-item self-report measure to assess participants’ depressive symptoms. Respondents are instructed to choose which statement best describes how they felt during the past two weeks including today. Items are rated on a 4-point (0–3) scale, with total scores obtained by summing the ratings for all items. A BDI-II score of 14 or greater has been suggested as a cut-off point for identifying individuals at risk for clinical depression [30].The BDI-II is a reliable and valid measure [30,31] with acceptable internal consistency in the current study (Cronbach’s Alpha = 0.86).

### 2.3. Neuroimaging data acquisition

The neuroimaging data were collected in the Southwest University China Center for Brain Imaging using a 3.0 T Siemens Trio MRI scanner (Siemens Medical, Erlangen, Germany). A magnetization-prepared rapid gradient echo (MPRAGE) sequence was used to acquire high-resolution T1-weighted anatomical images (repetition time = 1 900 ms, echo time = 2.52 ms, inversion time = 900 ms, flip angle = 9°,resolution matrix = 256 × 256, slices = 176,thickness = 1.0 mm, voxel size = 1 × 1 × 1 mm³). During resting-state fMRI scanning, the subjects were instructed to lie down, close their eyes, and rest without thinking about a particular thing, but not to fall asleep. The 8-min scan of 242 contiguous whole-brain resting-state functional images was obtained using gradient-echoplanar imaging (EPI) sequences with the following parameters: slices = 32,repetition time (TR)/echo time (TE) = 2000/30 ms, flip angle = 90°,field of view (FOV) = 220 mm × 220 mm, and thickness/slice gap = 3/1 mm,voxel size 3.4 × 3.4 × 4 mm³.

### 2.4. fMRI Data Preprocessing

All fMRI data were preprocessed using SPM8 (http://www.fil.ion.ucl.ac.uk/spm) and a Data Processing Assistant for Resting State fMRI (DPARSF). We first discarded the first 10 EPI scans to suppress the equilibration effects and the remaining scans were slice timing corrected. Then, the data were realigned and normalized to a standard template (Montreal Neurological Institute) and resampled to 3 × 3 × 3 mm³. All fMRI time-series underwent spatial smoothing (8 mm Full Width Half Maximum FWHM), band-pass temporal filtering (0.01–0.1 Hz), nuisance signal removal from white matter and cerebrospinal fluid, and 6 rigid-body motion correction parameters. We carefully performed the following procedures to ensure data quality: 1) subjects with poor structural scans, or functional MRI data, making successful preprocessing, that is, normalization to Montreal Neurological Institute (MNI) space, difficult or impossible, or without complete demographic information, were excluded; and 2) for head movement, subjects were excluded with >10% displaced frames in a scrubbing procedure, or maximal motion between volumes in each direction >3 mm, and rotation about each axis >3°. See supplemental information for a detailed discussion of global mean signal regression and data scrubbing.

#### ROI definition.

Based on previous research [9,12,32] bilateral DMPFC and sgACC were selected as regions of interest (ROIs), and these ROIs were generated using Brainnetome_v1.01.1 (DMPFC = SFG7_6, sgACC = CG7_7). A unified segmentation procedure (SPM12) was used to estimate parameters relating individual anatomy to MNI space. The inverse normalization parameters were used to create subject specific (gray matter) ROIs in native space based on the MNI masks.

### 2.5. Resting-state functional connectivity analysis

The voxel-wise seed-based functional connectivity analyses were performed using DPABI toolbox (Yan et al., 2016) (http://rfmri.org/dpabi). The analyses were conducted using the defined ROIs (bilateral DMPFC and sgACC) as seed regions to examine their whole-brain functional connectivity patterns. Pearson’s correlations coefficients between the time series of each seed region with voxels of the rest of the brain were then calculated for each ROI. A Fisher transformation was then used to convert these voxel-wise Pearson correlation coefficients into whole-brain z-values for each participant to conduct second-level analyses in SPM12. Combined the parameter estimate values of FC and behavioral data, multiple regression was conducted within SPM12, and rumination was included as independent variable, age, gender and mean Framewise Displacement (FD) values as covariates. FD was computed by first deriving the temporal differences between consecutive time points for the three translational and three rotational motion parameters. Rotational displacements were converted from radians to millimeters, assuming a spherical head model with a 50 mm radius, following the method described by Power et al. (2012) [33]. The mean FD across all time points was then calculated to characterize the participant’s overall head motion during the scan. To identify significant clusters, all tests were corrected for multiple comparisons using cluster-level FWE corrected at p < 0.05 and voxel level uncorrected at p < 0.001.

### 2.6. Statistical analysis

In order to examine whether functional connectivity that significantly correlated with rumination could predict individual BDI scores, regression analysis was conducted. However, as a result of the BDI scores does not satisfy the precondition of linear regression analysis, namely, the dependent variable must be normal distribution. Thus, we decided to transform the BDI scores as a dichotomous variable according to the criterion of Beck et al. [30].To be more specific, we labeled the BDI scores that less than 14 as 0 (N = 103), which represent healthy group, and that be equal or greater than 14 as 1 (N = 648), suggesting that these people may be at risk for depressive symptoms. After that, a logistic regression analysis was performed with SPSS22.0, in which parameter estimates extracted from the significant functional connectivity were included as independent variables respectively, BDI dichotomous values as dependent variable, and age, gender, mean FD_power as covariates. The significance level was set as 0.05.

## 3. Results

### 3.1. Behavioral result

**Fig 1:**
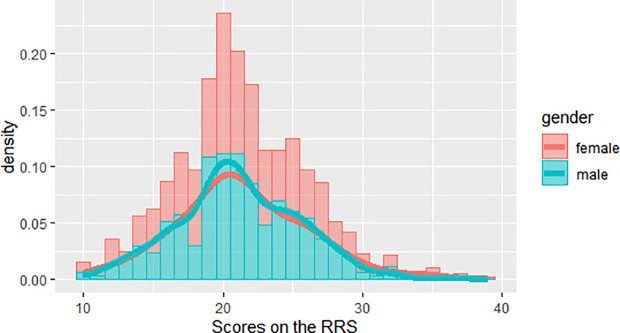
The score distribution of rumination.

In the present study, 751 non-clinical healthy participants were included. The kurtosis (0.42) and skewness (0.31) of SRRS scores fell within the range of −1 to +1, indicating that the data followed a normal distribution [34]. The score distribution of rumination is shown in Fig 1. Additionally, SRRS scores were significantly and positively correlated with BDI scores (*r* = 0.314, *p* < 0.01). The correlation between rumination and depression was significant for both females (*r* = 0.266, *p* < 0.01) and males (*r *= 0.383, *p* < 0.01). Descriptive statistics for all variables, including SRRS and BDI scores, are displayed in Table 1.

**Table 1:** Demographic and psychological variables.

Variables	Mean	SD
Sex (female/male)	420/331	
Age	19.61	1.48
SRRS	21.29	4.68
BDI	6.68	5.97

Note. SRRS: Short Ruminative Response Scale; BDI-II: Beck Depression Inventory-II.

### 3.2. fMRI result

#### 3.2.1. Trait rumination, depressive symptoms, and functional connectivity.

**Fig 2:**
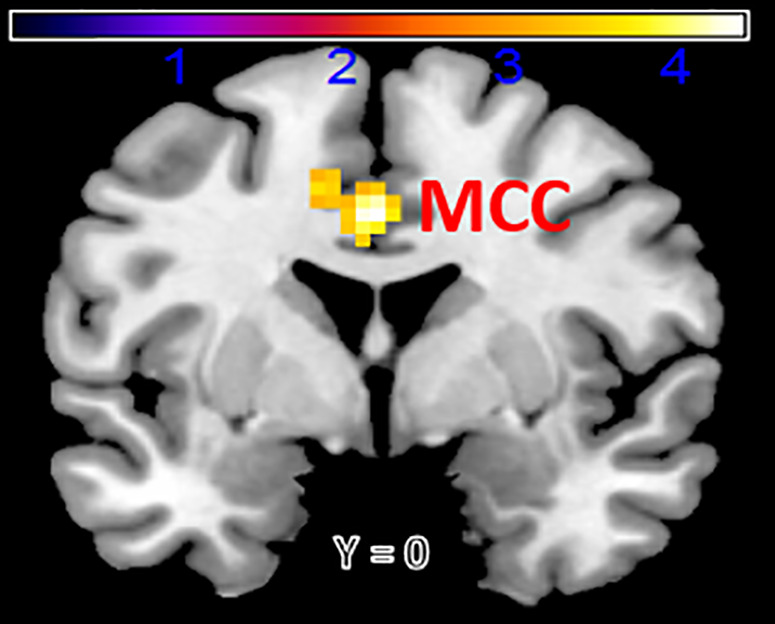
Functional connectivity between left dorsal-medial prefrontal cortex (DMPFC) and mid-cingulate cortex (MCC) associated with rumination.

**Fig 3:**
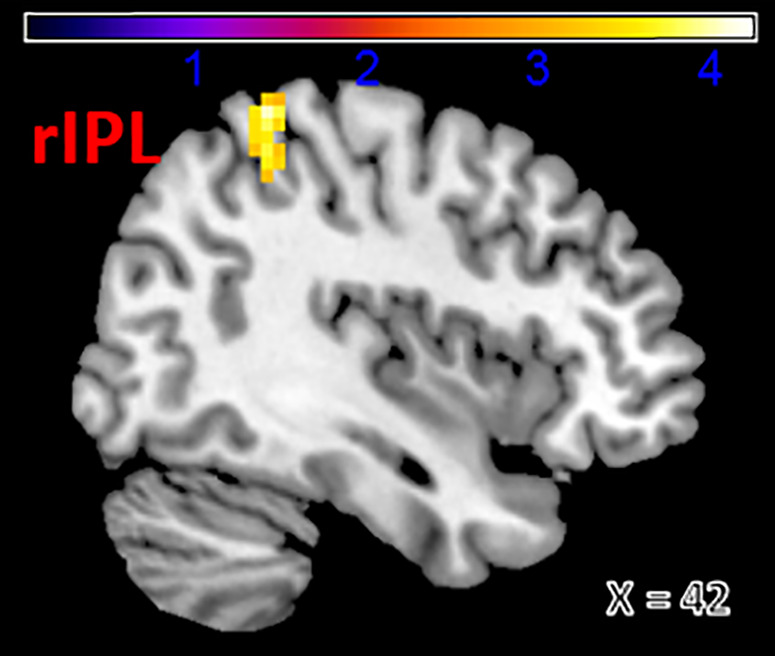
Functional connectivity between left dorsal-medial prefrontal cortex (DMPFC) and right Inferior Parietal Lobule (rIPL) associated with rumination.

To examine functional connectivity using the left dorsomedial prefrontal cortex (DMPFC) as a seed region in relation to trait rumination, a multiple regression analysis was conducted in SPM12. The results showed that rumination was significantly and positively correlated with functional connectivity between the left DMPFC and the left mid-cingulate cortex (MCC_L) (*Z* = 4.37, MNI coordinates: x = 0, y = 0, z = 39) and between the left DMPFC and the right Inferior Parietal Lobule (IPL_R) (Z = 4.22, MNI coordinates: x = 42, y = −42, z = 63) after the family-wise error (FWE < 0.05) corrected (see Table 2, Figs 2 and 3), indicating that greater levels of rumination was associated with stronger functional connectivity related to DMPFC.

**Table 2:** Regions showing a main effect of rumination.

seed	hemi	region	BA	X	Y	Z	k	Peak Z
DMPFC	Right	IPL	40	42	−42	63	50	4.22
	Left	MCC	/	0	0	39	49	4.37

Note. BA refer to Brodmann area; x, y, and z refer to MNI coordinates; k refers to the number of voxels in each significant cluster; Peak Z refers to peak activation level in each cluster; p refers to FWE-corrected significance level; IPL = Inferior parietal lobe; MCC = Mid-cingulate cortex.

#### 3.2.2. Functional connectivity showed a significant relationship with depressive symptoms.

Based on the above analysis results, we extracted the parameter estimations of signal intensity of the IPL and MCC. Using these estimates along with behavioral data, a logistic regression analysis was conducted in SPSS 22.0. In this analysis, the two parameter estimates were included as independent variables, while BDI dichotomous values served as the dependent variable. Age, gender, and mean FD_power were included as covariates.. The results showed that the positive functional connectivity between the left DMPFC and MCC significantly predicted depressive symptoms (*B* = 1.242, *S.E.* = 0.52, *Wald* = 5.715, *p* = 0.03, *Exp (β)* = 3.464), indicating that greater depressive symptoms were related to increased DMPFC connectivity with the left MCC. However, functional connectivity between DMPFC and the right IPL was not relative to depressive symptoms remarkably (*B* = 0.7, *S.E.* = 0.661, *Wald *= 1.123, *p* = 0.289, *Exp (β)* = 2.014).

## 4. Discussion

Using seed-based functional connectivity analysis in a relatively large sample, the current study examined the neural correlates of trait rumination and its potential relevance to depressive symptomatology in late adolescents. First, increased functional connectivity between the left DMPFC and MCC was positively associated with rumination scores. Second, a similar positive association was observed between the left DMPFC and the right IPL. Third, this DMPFC-MCC functional connectivity was associated with depressive symptoms, as assessed by dichotomized BDI scores.

### 4.1. Increased resting-state connectivity involving the DMPFC

We found that functional connectivity between the left DMPFC and MCC was positively related to rumination tendency. The DMPFC, a core node of the DMN’s DMPFC subsystem, has been linked to mentalizing and introspective processes such as monitoring one’s internal state [32,35,36]. Prior studies have have reported hyperactivity in this region among individuals with elevated rumination or depressive symptoms [24,26,37]. Moreover, in a meta-analysis concerning the rumination vs. distraction/control contrast, rumination-related hyperactivation was principally existed in the core and the DMPFC subsystems [12]. The mid-cingulate cortex (MCC) has been implicated in the control of goal-directed behaviors, including initiating movements, body-centered actions, and orienting responses such as exploratory eye and head movements. It also plays a role in processing vestibular and somatosensory information [38]. Supporting its relevance to rumination, an fMRI study found that MCC activity (Brodmann area 24) was positively associated with RRS scores when participants viewed sad facial expressions [39]. Moreover, voxel-based morphometry studies have shown that rumination is negatively correlated with gray matter volume in the bilateral MCC [40]. These structural and functional findings may help explain the observed abnormal functional connectivity between the DMPFC and MCC in individuals with higher trait rumination.

We also observed enhanced functional connectivity between the left DMPFC and right IPL, which was similarly associated with greater rumination. This may reflect altered connectivity within the default mode network, consistent with prior research [10,19,20,41,42]. Previous neuroimaging and lesion studies have shown that the inferior parietal lobule (IPL) is involved in sustaining and regulating attention over time [43,44], as well as detecting salient novel events [45]. One key feature of rumination is its self-perpetuating nature [46], which may be linked to prolonged attention on distressing thoughts. Heightened IPL activity may thus contribute to this sustained attentional focus, exacerbating ruminative thinking. Supporting this, an fMRI study found that increased IPL functional connectivity was associated with greater ruminative tendencies, particularly when comparing individuals with higher versus lower levels of positive affect [47]. Similarly, Burkhouse et al. [13] reported that the IPL was consistently recruited during rumination across both remitted depressed adolescents and healthy controls, and that greater IPL activation correlated with higher self-reported rumination and depressive symptoms. Together, these findings suggest that co-activation between the DMPFC and IPL may reflect a neural mechanism by which individuals sustain internally focused, self-referential processing, hallmarks of trait rumination.

### 4.2. DMPFC–MCC connectivity and depressive symptoms

Although we examined depressive symptoms using a dichotomized BDI measure due to its distribution, our findings suggest that individuals with higher functional connectivity between the DMPFC and MCC tend to report more depressive symptoms. While this pattern is suggestive of a potential neural correlate of risk for depression, it is important to emphasize that these findings are correlational and do not imply causality or predictive power in a clinical sense. Rather, the observed connectivity may contribute to our understanding of vulnerability mechanisms underlying depressive symptomatology in adolescents.

### 4.3. Limitations and conclusion

Several limitations warrant mention. First, the cross-sectional nature of the study precludes conclusions about causality. Longitudinal studies are needed to examine whether altered DMPFC connectivity precedes or follows changes in rumination and mood symptoms. Second, our sample comprised healthy college students, which may limit generalizability to clinical populations. Third, rumination was treated as a unidimensional construct, whereas prior research suggests it includes distinct subtypes such as brooding and reflection [29], or abstract and concrete rumination [48]. Future studies should explore these dimensions separately to capture more nuanced relationships. In summary, our findings support the relevance of DMPFC-MCC and DMPFC-IPL connectivity to ruminative tendencies and depressive symptoms in adolescents. These connectivity patterns may serve as neural markers of risk but should be interpreted with caution due to the study’s design and sample characteristics. Continued research is needed to clarify their role in the development and maintenance of depressive symptoms.
